# A Centrifugal Microfluidic Platform That Separates Whole Blood Samples into Multiple Removable Fractions Due to Several Discrete but Continuous Density Gradient Sections

**DOI:** 10.1371/journal.pone.0153137

**Published:** 2016-04-07

**Authors:** Scott T. Moen, Christopher L. Hatcher, Anup K. Singh

**Affiliations:** 1 Sandia National Laboratories, Livermore, California, United States of America; 2 University of Texas Medical Branch, Galveston, Texas, United States of America; The Ohio State University, UNITED STATES

## Abstract

We present a miniaturized centrifugal platform that uses density centrifugation for separation and analysis of biological components in small volume samples (~5 μL). We demonstrate the ability to enrich leukocytes for on-disk visualization via microscopy, as well as recovery of viable cells from each of the gradient partitions. In addition, we simplified the traditional Modified Wright-Giemsa staining by decreasing the time, volume, and expertise involved in the procedure. From a whole blood sample, we were able to extract 95.15% of leukocytes while excluding 99.8% of red blood cells. This platform has great potential in both medical diagnostics and research applications as it offers a simpler, automated, and inexpensive method for biological sample separation, analysis, and downstream culturing.

## Introduction

Separating blood components from whole blood is a necessary step in many clinical and research protocols. Conventionally, this process involves some variation of density gradient centrifugation to separate the components based on their specific sedimentation rates. Experimentally, extrication of a targeted constituent may be used for microRNA analysis [1] [2], production of peripheral blood monocytes cultures for infection assays [3] [4], cultivation of immune cells for chemotaxis assays [5] [6], and production of plasma cells from peripheral blood memory B cells [7]. The density gradient centrifugation process requires trained personnel and a fair amount of dexterity to load density layers and accurately extract the desired blood fractions. In addition, a relatively large amount of blood is needed (typically 1–10 mL [8][9]) to observe the discrete band of leukocytes for extraction. However, in most clinical applications, it would be advantageous to have the option of using smaller amounts of blood to perform the analysis.

We have developed a novel, centrifugation-based microfluidics platform for small-scale blood component separation. The platform consists of a single-use, “CD-like” laser-cut plastic disk with channels that contain laser-ablated capillary valves traversing radially, which keeps each density section spatially separated until the centrifugation step. Additionally, a microprocessor controlled centrifugal actuator controls acceleration, deceleration, and time under centrifugation to separate the assay components in the disk. Our system offers a number of advantages over existing centrifugal platforms to sort blood components. First, it is a simpler and less expensive approach to fractionate blood. Unlike other centrifugal CD platforms, ours does not require antibodies [10] [11], fluorophores [10], lasers [11] [12], syringe pumps [10], or magnetic beads [12]. Our system simply requires the equipment to drive a small dc motor, and the establishment of density gradients using inexpensive chemicals (e.g. sucrose) to separate cells based on sedimentation through the density medium. A few reported platforms are only able to extract a single cell type via affinity-bead systems or single density gradient [10] [13]. Our system also differs in that samples can be obtained directly from each density section, allowing the selective removal of multiple viable cell types depending on the density separation characteristics of the setup.

To exemplify the direct microscopic analysis of separated cells, we also adapted Wright-Giemsa staining for use in our microfluidic platform. Hematological microscopy, specifically the Wright stain, has remained greatly unchanged since its inception in 1891 [14] and is frequently used to determine phenotypical microscopic observations in diagnosis of pathologies such as infections and leukemia. The blood smear technique used to prepare whole blood for staining and microscopy requires training and experience to create a consistent homogenous smear of sufficient thickness for optimal staining. By adapting this process to the microfluidic platform, we have greatly reduced the variability, minimized chances of infection during sample preparation, improved the concentration of similar cell types, and simplified the staining process.

The benefits inherent to our system include sterile processing, small sample volume, and minimal ancillary equipment, such as pumps or centrifuges. These advantages make our design amenable to many applications in diagnostics and research that require blood fractionation.

## Results and Discussion

### Density centrifugation and mechanical setup

**Fig 1:**
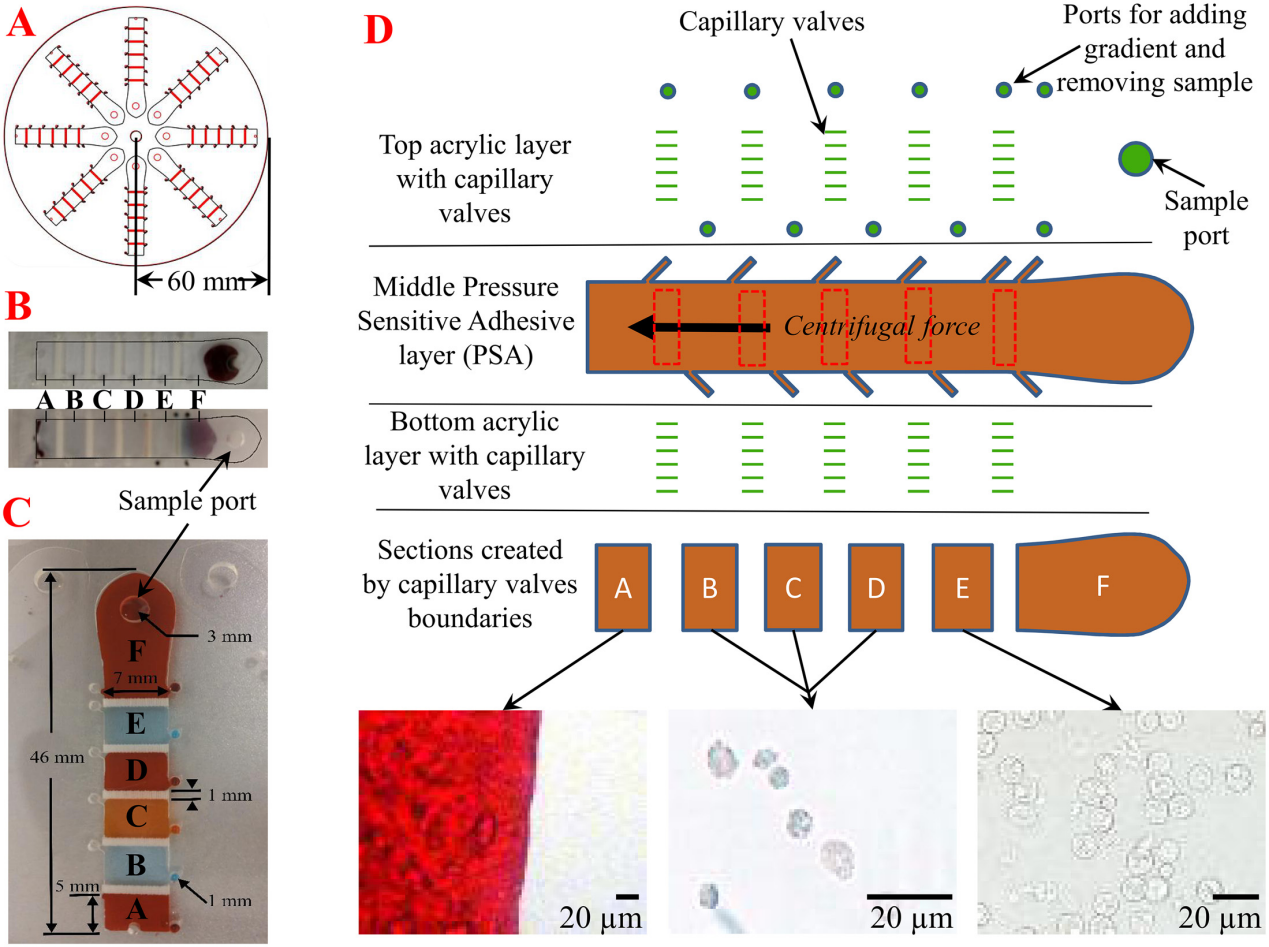
Physical configuration of the microfluidic disk. (A) Each sample disk has eight lanes with a 60 mm radius. (B) Images of a loaded (top) and post-centrifugation (bottom) sample lane. (C) Sample lane shown with alternating colors of food coloring to demonstrate the ability of the capillary valves to keep sections separated prior to centrifugation. (D) Schematic channel view with examples of partition constituents following blood separation. Partition A is where the majority of red blood cells settle. Partitions B, C, and D are relatively cell-free areas, while partition E is contains the vast majority of leukocytes. Images taken on Nikon TI inverted microscope (40X DIC) and auto white balanced using the NIS Elements AR 4.12.01 software.

The assay disk was fabricated as described in the Experimental Section and the setup is shown in Fig 1. In the current iteration, the disk is divided into eight lanes, each consisting of five discrete density sections (A, B, C, D, and E) with two ports for each. These ports allow the user to introduce density gradient, while the opposing hole allows the displaced air to release. Post-centrifugation, these ports allow the removal of sample from a section by opening the appropriate holes. An extra outlet was added to section A to remove potentially packed cells at the end of the lane depending on the separation being performed. Section F is where the sample is introduced and maintained until centrifugation. These sections are defined by laser-ablated troughs on the luminal surfaces of both layers of plastic that act as capillary burst valves and keep the gradients separated while loading. These ablated luminal surfaces form a roughly perpendicular valve edge that “pins” the meniscus of the contained fluid (in this case the density gradient) until the applied force (centrifugal) surpasses its holding power. This is called the burst threshold. We used the Young-Laplace equation, to approximate this value for system design:

$$\;P_{h}=-2\sigma \text{cos}\;\theta_{A}\left(\frac{1}{h}+\frac{1}{w}\right)$$ (1)

In this equation, ***P***_***h***_ is the pressure difference across the meniscus [15]. ***σ*** is the surface tension of the liquid (blood [16]), and ***θ***_***A***_ is the advancing contact angle (blood on acrylic [17]), while *h* and *w* are the height and width of the channel, respectively.

Once the pressure difference that “pins” the meniscus is known, it can then be substituted in the following formula to determine the RPM needed to overcome this burst threshold and allow separation of the sample:

$$P_{A}=.5\rho \omega^{2}\left(r_{p}{}^{2}-r_{d}{}^{2}\right)$$ (2)

In this formula, ***ρ*** is the liquid density (blood [18]), and ***ω*** is the constant angular velocity (consequently RPM), while ***r***_***p***_ and ***r***_***d***_ are the proximal and distal radii of the liquid, respectively.

In real-world application, these valves allow the set-up of a density gradient on a horizontal platform, and ameliorate perturbations induced by the loading process. However, the valves are easily overcome when centrifugal force is applied. We adjusted our system to theoretically respond to 100 RPM (1 RCF [relative centrifugal force]) so that weak centrifugal force can be employed for the separation of biological components characterized by low specific density, such as platelets.

**Fig 2:**
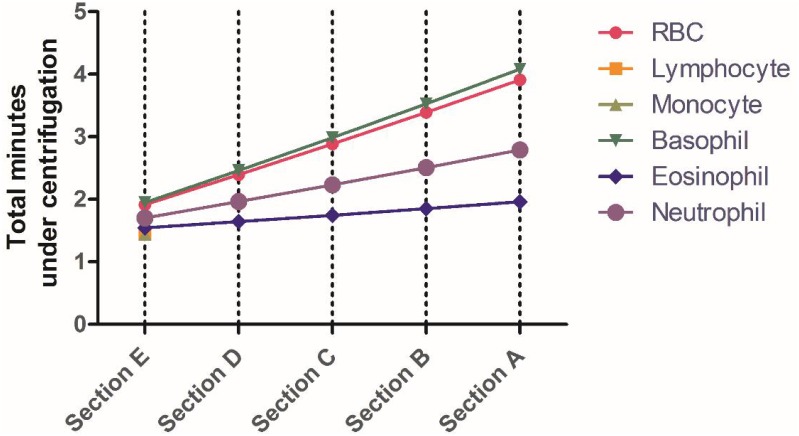
Total time of centrifugation for our target cell as predicted by Eq 3. Using the Histopaque^®^ 1077 gradient, with an angular velocity of 500 RCF, we can predict the time it takes for RBCs, lymphocytes, monocytes, basophils, eosinophils, and neutrophils to reach various sections in our sample lane. Section F is where the sample is introduced and section A is most distal.

Upon centrifugation, sample constituents experience centrifugal force and travel radially outward through these discrete density sections. The time required to fractionate different cell types is calculated by using the equation below:

$$\;T=\frac{9\eta \text{ln}\left(\frac{r_{d}}{r_{p}}\right)}{8\pi^{2}\omega^{2}R^{2}\left(\rho -\rho^{{}^{\circ}}\right)}+\frac{2\left(t_{a}+t_{d}\right)}{3},$$ (3)

where η is the viscosity, and $\frac{r_{d}}{r_{p}}$ is the ratio of distal and proximal distance from the axis of rotation. ω is the angular velocity in rev/s, *R* is the effective particle radius, ρ is the cell density, ρ° is the density of the gradient, ***t***_***a***_ is acceleration time, and ***t***_***d***_ is the time of deceleration [19]. Each section in the disk has a discrete density medium. Therefore, we add the times for each density section to determine the overall time for centrifugation, and consequently, the location of various cell types. Fig 2 shows how this equation is used to determine the total time, at 500 RCF and in Histopaque^®^ 1077, for a specific cell type to reach a specific section in our sample lane. Lymphocytes and monocytes are not predicted to migrate because of their low specific densities and relative size. We used these calculations as a starting point for experimental pilot studies with each application on our device.

### Human blood leukocyte enrichment

In order to exemplify our system’s ability to sort cells for removal, we chose to mimic the commonly employed buffy coat density separation. The separation is frequently used to isolate living leukocytes for further subculture from whole blood samples, or to identify intracellular pathogens. Traditionally, this separation is performed with a centrifuge with a swinging-bucket rotor, and the blood is layered over a carefully constructed density gradient consisting of one or multiple layers. This technique requires a relatively large amount of blood because of the large size of the tubes and the need for the discrete band of leukocytes to be discernible via the naked eye for successful extraction. Once centrifugation is finished, the traditional method requires careful aspiration of the lighter density layers until the target layer is reached. Finally, the sample should be verified by creating a slide for microscopy.

**Fig 3:**
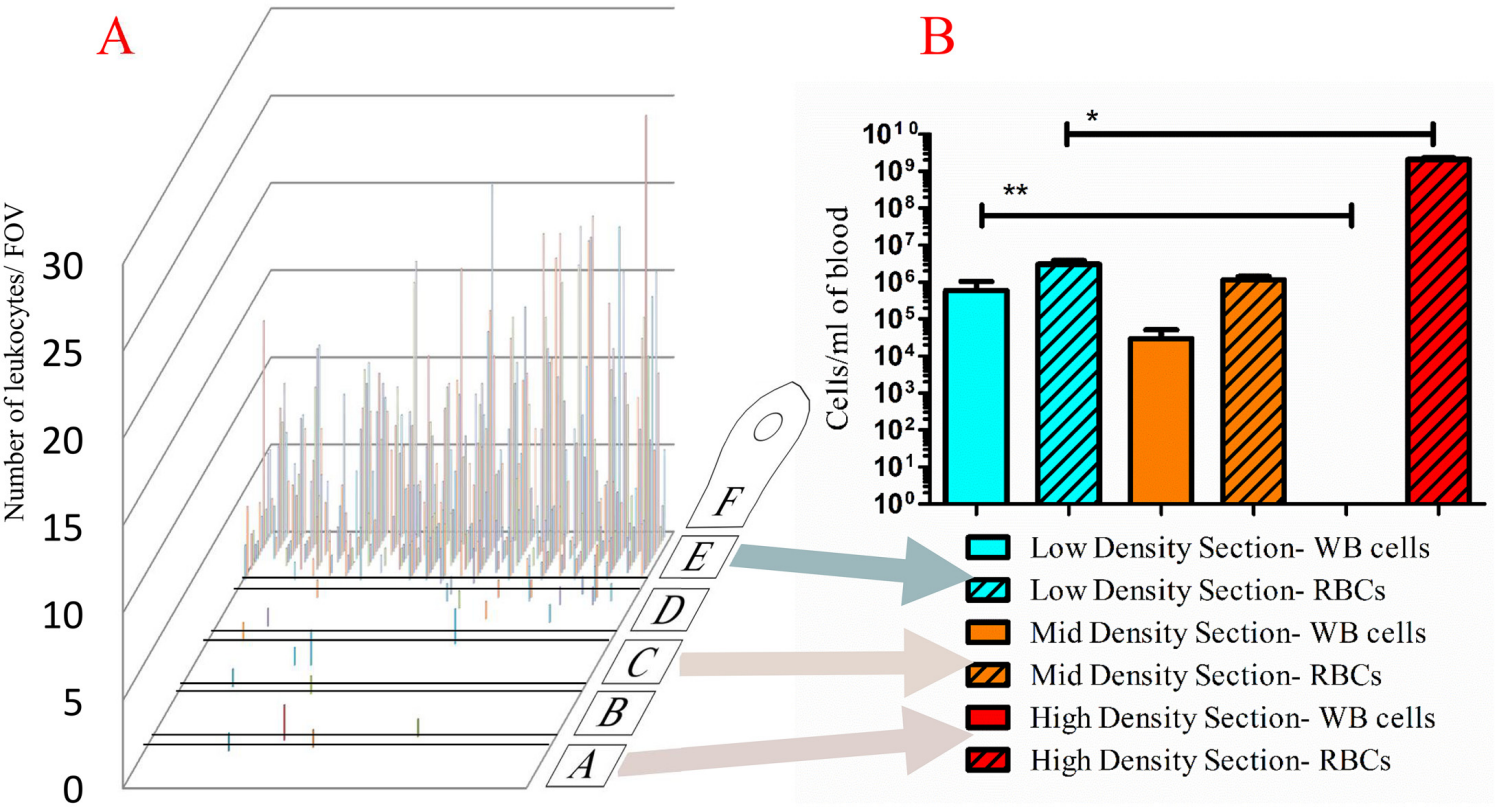
Location of blood components post-centrifugation. (A) Location and quantity of leukocytes after 4-min separation in density media as counted on disk via microscopy (figure is representative of n = 3). Background image denotes the orientation of the lane and location of the capillary valves. (B) The number of red blood cells and leukocytes extracted from section E, section C, and section A and counted with a Neubauer chamber. Results from three assay disks are shown to demonstrate disk-to-disk reproducibility. (WBCs are significantly enriched in section E versus section A, two-way ANOVA ** = p<0.05; RBC’s are significantly enriched in section A versus section E, * = p<0.05.)

**Fig 4:**
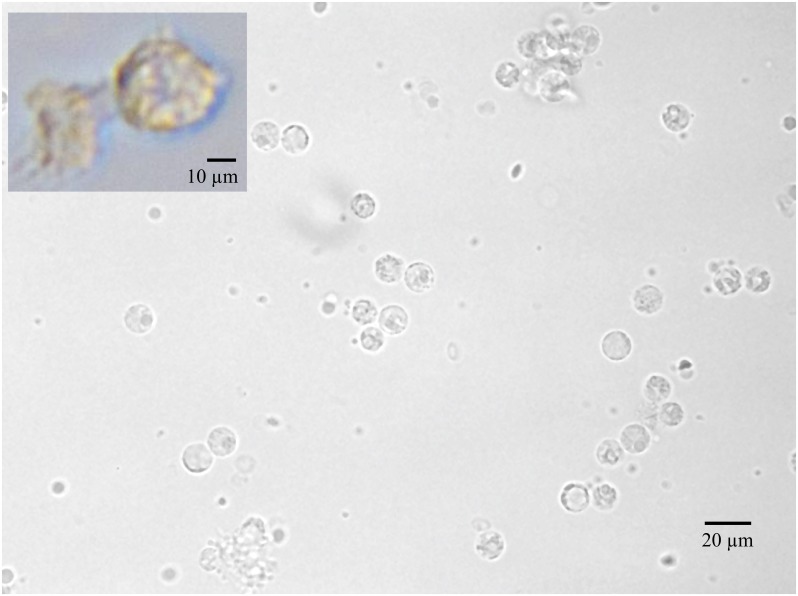
Typical field of view (FOV) for cell quantitation (Nikon TI 40X DIC). Images of the disk can be used for direct cell enumeration. A TIFF format picture was taken of each FOV using a Nikon TI inverted microscope (1,800 FOVs/sample lane. This photo shows one such FOV in section E, where the predominant visualized cell type is the leukocyte. Inset shows a monocyte that was removed from a disk post- centrifugation and cultured until a macrophage phenotype was seen. Images taken on Nikon TI inverted microscope (40X DIC) and auto white balanced using the NIS Elements AR 4.12.01 software.

As there are roughly 5 x 10^6^ RBCs/μL [20] versus 4.5–10 x 10^3^ leukocytes/μL [21] in whole blood, leukocyte enrichment is required to increase the signal-to-noise ratio for microscopic analysis, or for extraction of a pure leukocyte cell population. We used our microfluidic disk-based density centrifugation setup to perform this enrichment by using the traditional density media and RCF, to determine whether our platform would perform in a manner that was qualitatively similar to that of traditional centrifugation. First, we exploited one of our method’s unique features, the ability to count cells directly on the disk by microscopy. Fig 3A shows the location and quantity of leukocytes counted on the disk, with the majority of cells migrating to the proximal section (section E). Although the RBC sedimentation is not able to provide 100% separation, our method provides significant enrichment, as seen in a typical field of view (FOV) in Fig 4. Next, we removed cells from each section with a pipette and counted the cells using a Neubauer chamber. Fig 3B depicts the quantities of red blood cells and leukocytes extracted from various sections of the assay lane. Sections B, C, and D had characteristically the same cell types for this assay so we counted only cells from section C as a representative of these sections. On average, 99.8% of the red blood cells were excluded from the proximal section, while 95.15% of the leukocytes were retained.

Sedimentation rates follow Stokes’ Law, and, since all other variables are the same in each microfluidic channel, the particle size and density determine sedimentation rates. Table 1 lists the radii and specific density of blood components. Red blood cells have a smaller radius than leukocytes, with a slightly higher density, and consequently should sediment at the same rate as the leukocytes. However, RBCs frequently agglutinate, which creates particles with a larger effective radius and faster sedimentation rate than single RBCs.

**Table 1:** The specific densities and effective radii of common blood components.

*Blood cell type*	*Specific density(g/mL)*	*Effective radius (μm)*
Monocyte	1.067–1.077[22]	6–7.5[23]
Lymphocyte	1.073–1.077[22]	3–6[23]
Basophil	1.072–1.078[24]	4.5–5[23]
Neutrophil	1.08–1.09[25]	6–7.5[23]
Eosinophil	1.09–1.1[25]	6–7.5[23]
RBC	1.098[26]	2.63 (oblate spheroid)

### Modified Wright-Giemsa staining method

The modified Wright-Giemsa stain generally consists of two dyes, a cationic methylene blue and an anionic eosin, that differentially stain blood cells. It is a ubiquitous method of analyzing blood for disease and infection but it is not without shortcomings. The protocol is manual, time consuming, and performed in an open environment, making it subject to user variability and a potential source of infection. The miniaturized centrifugal platform can overcome most of these shortcomings. Our method uses the same reagents as the traditional protocol; however, by mixing the cells and staining them in a microcentrifuge tube, our method facilitates complete cell penetration. Staining of cells prepared via the glass slide blood smear technique only allows for surface penetration of the stain. Additionally, the traditional method uses phosphate-buffered saline (PBS) to wash cells that are fixed on a glass slide in order to remove unbound dye from the blood smear. However, this process can add variability through the accidental washing-off of cells or by varying dye incubation times. In our method, the wash is operator-independent and standardized from run to run, as stained cells pass through PBS contained in section E during centrifugation. Unbound dye has a significantly smaller effective radius and density relative to the cells, thus, it remains outside of the assay area.

**Fig 5:**
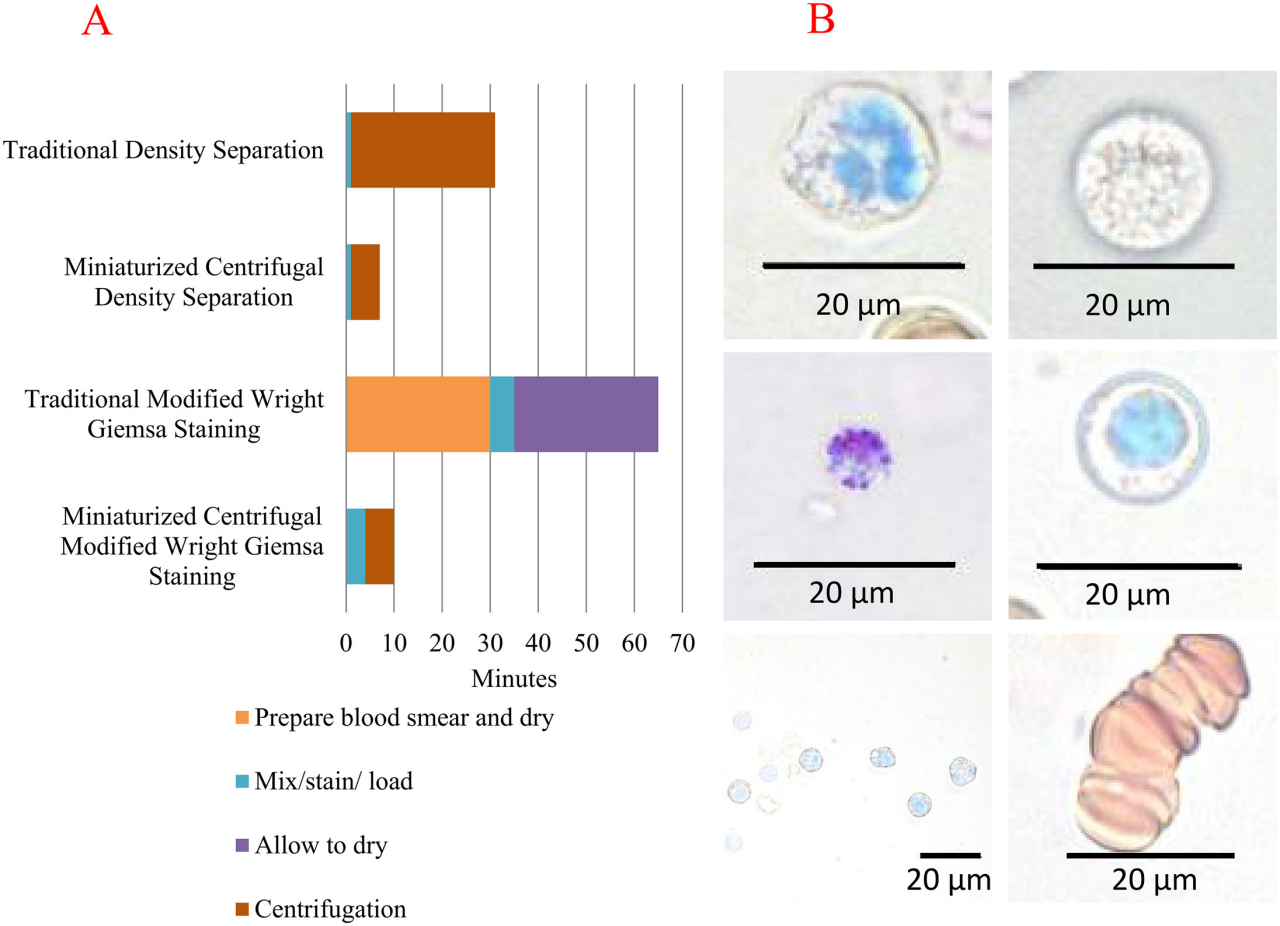
Sample processing time for our miniaturized centrifugal platform versus traditional methods. (A) Comparison of protocol times for our miniaturized centrifugal platform and their corresponding traditional methods, in minutes. In both methodologies, buffy coat separation and modified Wright-Giemsa stain, the miniaturized centrifugal protocols were significantly shorter. (B) Modified Wright-Giemsa stained blood cells. Clockwise from bottom left: enriched cell FOV, basophil, neutrophil, monocyte, lymphocyte, and Rouleaux formation. All images were taken from section E with the exception of the Rouleaux formation which was taken from section B. Images were captured using a Nikon TI 40X DIC and auto white balanced using the NIS Elements AR 4.12.01 software.

We compared the time requirements of our miniaturized centrifugal platform with the traditional counterparts for both leukocyte fractionation and Wright-Giemsa staining in Fig 5A. Our miniaturized centrifugation platform uses a shorter centrifugation path, roughly half of a 50 ml conical, which considerably shortens the overall protocol time. In the case of the modified Wright-Giemsa stain, the microfluidic platform shortened the procedural time due to lack of many of the preparatory steps needed in the traditional method.

Finally, in our system, the closed sample chambers reduce the risk of laboratory-acquired infection. After the initial incubation of dye and whole blood in a microcentrifuge tube, the entirety of our protocol is conducted in a sealed apparatus. Traditional methodologies, however, require samples that possibly contain infectious agents to be manipulated and assayed in an open environment (using glass slides). Fig 5B demonstrates the success of our miniaturized centrifugal modified Wright-Giemsa staining protocol in staining various immune cells from blood. However, unlike the smear method, our technique places cells at more than one Z plane, so care must be taken during imaging. An additional benefit of our system (Fig 1C) is that similar cell types, having similar sizes and specific densities, sediment at approximately the same velocity, and therefore cells of interest can be more readily located.

Another example of a clinically relevant target revealed in our Wright-Giemsa staining, Rouleaux formation, can be seen in Fig 5B and is important for the diagnosis of diabetes, cancer, and some infections. This formation is artificially induced by our choice of gradient, Histopaque 1077, which was selected for its cell separation characteristics. However, the flexibility of our system would allow gradient and buffers adjustments for such a detection.

## Experimental Section

### CD fabrication

A Universal Laser Systems 60 watt, 6.60 VLS laser was used to cut the layers of the CD from CAD EPS files. The top layer was cut from 1.524 mm extruded acrylic sheets, while the bottom layer was cut from 0.127 mm polycarbonate film to accommodate the minimal working distance of a 60X objective. Both layers were purchased from McMaster-Carr. A double-sided adhesive (3M 8153LE), consisting of a 0.05 mm polyester carrier layer sandwiched between a 0.28 mm and 0.34 mm adhesive layer, was used to create the channel layer and hold the two plastic layers together. After sonicating the plastic disk layers in distilled water, they were placed in a HEPA drying chamber. The plastic layers sandwiching the PSA layer were aligned and compressed in a roller press (Jack Richeson & Co Inc.). Finally, the input and outputs were covered with scotch tape to prevent microbial or gross contamination until the disk was used.

The disk contains eight separate lanes consisting of five discrete gradient sections, each 5 x 7 mm, separated by microfluidic capillary valves, as seen in Fig 1C. These capillary valves are 1 mm x 7 mm wide and etched into acrylic to create a density gradient boundary between each section that can be surpassed upon centrifugation. Each gradient section holds approximately 12 μL of media and is flanked by diagonally situated ports to maximize the amount of fluid that can be administered or removed. The sample is administered through a 3 mm hole located at the proximal portion of lane F.

### CD actuator and containment

A 9-volt generic DC motor was driven by a L293D H-bridge chip which received pulse width modulation from an Arduino UNO Rev3 microprocessor. We avoided a simple direct drive of the motor in order to create a program for controlling acceleration, run time, and deceleration, which ameliorated unwanted perturbation of sample and gradient. The CD accelerates at 500 RCF/min until 500 RCF is reached and is maintained for 4 minutes, at which time deceleration occurs at 322 RCF/min. By using a stroboscope, we calculated a PWM-to-RPM correlation with a loaded disk so that our code could attain the proper control without expensive feedback mechanisms.

The motor, spindle, and sample CD are contained in a 12.7 mm thick, clear acrylic box with 152.4 x 152.4 x 101.6 mm dimensions. The hinged top is encircled by a rubber gasket to contain potential aerosols. Samples were analyzed in a BSL-2 Biosafety cabinet while following proper UTMB biosafety protocols.

### Blood density centrifugation

We purchased 10 mL of human blood in a K_2_EDTA tube (tested for human blood-communicable pathogens) from Innovative Research and it was stored at 4°C until use. Prior to each experiment, the blood was slowly oscillated to homogenize the cells, and then 10 μL was mixed with 10 μL of PBS. Concurrently, sections A, B, C, D, and E were filled with room-temperature Histopaque^®^ 1077. Next, 5 μL of the diluted blood was added to the sample port in section F, all of the ports were then sealed with Microseal^®^C PCR film, and the centrifugation cycle started. The disk was then analyzed via 40X bright-field illumination on a Nikon TI inverted microscope to visualize the band of leukocytes. The Institutional Review Board at the University of Texas Medical Branch waived the approval process for our use of human blood as it was purchased with no identifying patient information.

### Modified Wright-Giemsa stain

We purchased 10 mL of EDTA-treated human blood (tested for human blood-communicable pathogens) from Innovative Research and maintained it at 4°C until use. Prior to each experiment, the blood was carefully oscillated to homogenize the cells, and then 10 μL of this blood was slowly pipetted with 2 μL of Sigma Modified Wright-Giemsa stain. Concurrently, sections A, B, C, and D were filled with room-temperature Histopaque^®^ 1077, while section E received PBS. After 2 min incubation, 8 μL of PBS was mixed with the sample. Immediately following another 2 min incubation, 10 μL of sample was added to the sample port in section F. All ports were covered with Microseal^®^C PCR film and the centrifugation cycle was started.

The disk was then analyzed via 40X bright-field illumination on a Nikon TI inverted microscope. An automated x-y sequence was initiated to cover every FOV for the channel. At each FOV, the Z was manually adjusted to the lowest layer of cells and a TIFF (1,800 FOVs/lane) was saved. Leukocyte counts were manually performed for each FOV and the order rearranged to fit the physical configuration of the channel for the purposes of presentation.

## Conclusions

While traditional centrifugation is a widely used technique for fractionation of whole blood, it is slow, laborious, and requires a relatively large blood volume. The miniaturized centrifugal platform presented here reduces the analysis time and the amount of blood needed. It also allows staining of blood cells with minimal human intervention and reduces the chance of accidental infection, as the disk is sealed prior to centrifugation. The adaptability of the platform allows for a variety of other procedures, such as assaying blood for parasites and extracting bacteria from complex biological matrices such as feces and organ tissue.

## Supporting Information

S1 Appendix Contains the raw data for Fig 2. (PZF)

S2 Appendix Contains the raw data for Fig 3A. (XLSX)

S3 Appendix Contains the raw data for Fig 3B. (PZF)

## References

[1] Pritchard CC, Kroh E, Wood B, Arroyo JD, Dougherty KJ, Miyaji MM, et al Blood cell origin of circulating microRNAs: A cautionary note for cancer biomarker studies. Cancer Prev Res. 2012;5: 492–497. 10.1158/1940-6207.CAPR-11-0370 PMC4186243 22158052

[2] Leidinger P, Backes C, Meder B, Meese E, Keller A. The human miRNA repertoire of different blood compounds. BMC Genomics. 2014;15: 474 10.1186/1471-2164-15-474 24928098 PMC4076980

[3] Fogeda M, Navas S, Martin J, Casqueiro M, Rodriguez E, Arocena C, et al In vitro infection of human peripheral blood mononuclear cells by GB virus C/Hepatitis G virus. J Virol. 1999;73: 4052–4061. Available: http://www.ncbi.nlm.nih.gov/pubmed/10196301 10196301 10.1128/jvi.73.5.4052-4061.1999 PMC104184

[4] Andre MC, Gille C, Glemser P, Woiterski J, Hsu H-Y, Spring B, et al Bacterial reprogramming of PBMCs impairs monocyte phagocytosis and modulates adaptive T cell responses. J Leukoc Biol. 2012;91: 977–989. 10.1189/jlb.0911474 22427683

[5] Traves SL, Smith SJ, Barnes PJ, Donnelly LE. Specific CXC but not CC chemokines cause elevated monocyte migration in COPD: a role for CXCR2. J Leukoc Biol. 2004;76: 441–450. 10.1189/jlb.1003495 15155777

[6] Colotta F, Sciacca FL, Sironi M, Luini W, Rabiet MJ, Mantovani A. Expression of monocyte chemotactic protein-1 by monocytes and endothelial cells exposed to thrombin. Am J Pathol. 1994;144: 975–985. 8178946 PMC1887349

[7] De Boer BA, Kruize YCM, Yazdanbakhsh M. In vitro production of IgG4 by peripheral blood mononuclear cells (PBMC): The contribution of committed B cells. Clin Exp Immunol. 1998;114: 252–257. 10.1046/j.1365-2249.1998.00732.x 9822284 PMC1905099

[8] Böyum A. Separation of leukocytes from blood and bone marrow. Introduction. Scand J Clin Lab Invest Suppl. 1968;97: 7 5707208

[9] Berthold F. Isolation of human monocytes by Ficoll Density Gradient Cntrifugation. Blut. 1981;400: 367–371. 10.1007/BF00320315 7332785

[10] Park JM, Lee JY, Lee JG, Jeong H, Oh JM, Kim YJ, et al Highly efficient assay of circulating tumor cells by selective sedimentation with a density gradient medium and microfiltration from whole blood. Anal Chem. 2012;84: 7400–7407. 10.1021/ac3011704 22881997

[11] Park JM, Kim MS, Moon HS, Yoo CE, Park D, Kim YJ, et al Fully automated circulating tumor cell isolation platform with large-volume capacity based on lab-on-a-disc. Anal Chem. 2014;86: 3735–3742. 10.1021/ac403456t 24641782

[12] Cho Y-K, Lee J-G, Park J-M, Lee B-S, Lee Y, Ko C. One-Step Pathogen Specific DNA Extraction from Whole Blood on a Centrifugal Microfluidic Device. TRANSDUCERS 2007–2007 International Solid-State Sensors, Actuators and Microsystems Conference. IEEE; 2007. pp. 387–390. 10.1109/SENSOR.2007.4300149

[13] Kinahan DJ, Kearney SM, Glynn MT, Ducrée J. Spira mirabilis enhanced whole blood processing in a lab-on-a-disk. Sensors Actuators A Phys. Elsevier B.V.; 2014;215: 71–76. 10.1016/j.sna.2013.11.010

[14] Cox FE. History of the discovery of the malaria parasites and their vectors. Parasit Vectors. 2010;3: 5 10.1186/1756-3305-3-5 20205846 PMC2825508

[15] Cho H, Kim H-Y, Kang JY, Kim TS. How the capillary burst microvalve works. J Colloid Interface Sci. 2007;306: 379–385. 10.1016/j.jcis.2006.10.077 17141795

[16] Rosina J, Kvasnák E, Suta D, Kolárová H, Málek J, Krajci L. Temperature dependence of blood surface tension [MANUSCRIPT]. Physiol Res. 2007;56 Suppl 1: S93–8. Available: http://www.ncbi.nlm.nih.gov/pubmed/17552890 17552890 10.33549/physiolres.931306

[17] Pitts KL, Abu-Mallouh S, Fenech M. Contact angle study of blood dilutions on common microchip materials. J Mech Behav Biomed Mater. Elsevier; 2013;17: 333–336. 10.1016/j.jmbbm.2012.07.007 23127640

[18] Vitello DJ, Ripper RM, Fettiplace MR, Weinberg GL, Vitello JM. Blood Density Is Nearly Equal to Water Density: A Validation Study of the Gravimetric Method of Measuring Intraoperative Blood Loss. 2015;2015: 7–9. 10.1155/2015/152730 PMC4590883 26464949

[19] Poppe LJ, Fredericks JJ, Hathaway JC. A computer program to calculate centrifugation parameters for sedimentation analyses. Comput Geosci. 1988;14: 541–545. 10.1016/0098-3004(88)90034-9

[20] Peter Klinken S. Red blood cells. Int J Biochem Cell Biol. 2002;34: 1513–1518. 10.1016/S1357-2725(02)00087-0 12379271

[21] Hoeboer SH, Groeneveld a. BJ. Changes in Circulating Procalcitonin Versus C-Reactive Protein in Predicting Evolution of Infectious Disease in Febrile, Critically Ill Patients. PLoS One. 2013;8: 24–27. 10.1371/journal.pone.0065564 PMC3675153 23762396

[22] Zipursky A, Bow E, Seshadri RS, Brown EJ. Leukocyte density and volume in normal subjects and in patients with acute lymphoblastic leukemia. Blood. 1976;48: 361–71. 1066173

[23] Meyer HS. Color Textbook of Histology. JAMA: The Journal of the American Medical Association. 2001 pp. 95–95.

[24] Raghuprasad PK. A rapid simple method of basophil purification by density centrifugation on Percoll. J Immunol. 1982;129: 2128–2133. 6181163

[25] Boyum A, Lovhaug D, Tresland L, Nordlie EM. Separation of leucocytes: Improved cell purity by fine adjustments of gradient medium density and osmolality. Scand J Immunol. 1991;34: 697–712. 10.1111/j.1365-3083.1991.tb01594.x 1749920

[26] Ratner BD, Hoffman AS, Schoen FJ, Lemons JE. Biomaterials science: an introduction to materials in medicine. Chemical Engineering. 2004.

